# Activation of the complement system in an osteosarcoma cell line promotes angiogenesis through enhanced production of growth factors

**DOI:** 10.1038/s41598-018-23851-z

**Published:** 2018-04-03

**Authors:** Hyungtaek Jeon, Seung Ro Han, Suhyuk Lee, Sang June Park, Joo Heon Kim, Seung-Min Yoo, Myung-Shin Lee

**Affiliations:** 1 Department of Microbiology and Immunology, Eulji University School of Medicine, Daejeon, 34824 South Korea; 2 Eulji Biomedical Science Research Institute, Eulji University School of Medicine, Daejeon, 34824 Republic of Korea; 3 Department of Pathology, Eulji University School of Medicine, Daejeon, 34824 South Korea

## Abstract

There is increasing evidence that the complement system is activated in various cancer tissues. Besides being involved in innate immunity against pathogens, the complement system also participates in inflammation and the modulation of tumor microenvironment. Recent studies suggest that complement activation promotes tumor progression in various ways. Among some cancer cell lines, we found that human bone osteosarcoma epithelial cells (U2-OS) can activate the alternative pathway of the complement system by pooled normal human serum. Interestingly, U2-OS cells showed less expression of complement regulatory proteins, compared to other cancer cell lines. Furthermore, the activated complement system enhanced the production of growth factors, which promoted angiogenesis of human endothelial cells. Our results demonstrated a direct linkage between the complement system and angiogenesis using the *in vitro* model, which suggest the complement system and related mechanisms might be potential targets for cancer treatment.

## Introduction

The complement system is not only an effector of innate immunity but also a participant in the adaptive immune response, inflammation, hemostasis, and more^1^. Recent findings indicate that complement activation in the tumor microenvironment may promote tumor growth and metastasis^2,3^. Upon complement activation, C3a or C5a modulate inflammation through chemotaxis, generation of radical oxygen species, and increasing vascular permeability. Apart from its role in the immune response, C5a seems to modulate a microenvironment for tumor progression. Pharmacological blockage of C5 and mice lacking C5aR resulted in decreased levels of TGF-β/IL-10 and impaired metastasis^4^. Reduced tumor growth and impaired angiogenesis were observed in a mouse model of epithelial ovarian cancer lacking C5aR signaling^5^. The formation of a membrane attack complex (MAC) in complement activation leads to structural pores within cell membranes, resulting in cell death by osmotic fluid shifts and cation influx^6^; however, many nucleated eukaryotic cells have defensive mechanisms against MAC-mediated destruction. This so-called sublytic MAC induces different effects on cells, including activation of the cell cycle, growth factor release, and protection from apoptotic cell death, among others^7–10^. Although MAC depositions have been reported in various cancer tissues, it is still unclear if complement activation is a friend or foe to cancer progression^2,3,7^. To investigate the effect and mechanism of the complement system on cancer progression, a convenient *in vitro* model would be invaluable. However, complement activation in cancer cells of most *in vitro* models is mediated by the antigen-antibody complex^10–12^, which requires not only expensive purified specific antibodies but also optimization processes to induce sublytic MAC. In this study, we present a novel *in vitro* model for complement activation in cancer cells using pooled normal human serum (NHS). NHS-treated human bone osteosarcoma epithelial cells (U2-OS) showed the activation of alternative pathway of complement system with sublytic levels of MAC, and conditioned media from complement-activated U2-OS cells significantly enhanced tube formation activity of human endothelial cells. Additionally, we found that this tube formation is mediated by the upregulation of secreted growth factors including FGF1 and VEGF-A through ERK phosphorylation. In this study, we demonstrate for the first time activation of the complement system in osteosarcoma cells using NHS, and the complement system’s impact on angiogenesis.

## Results

### Activation of complement system in U2-OS osteosarcoma cancer cells

**Figure 1:**
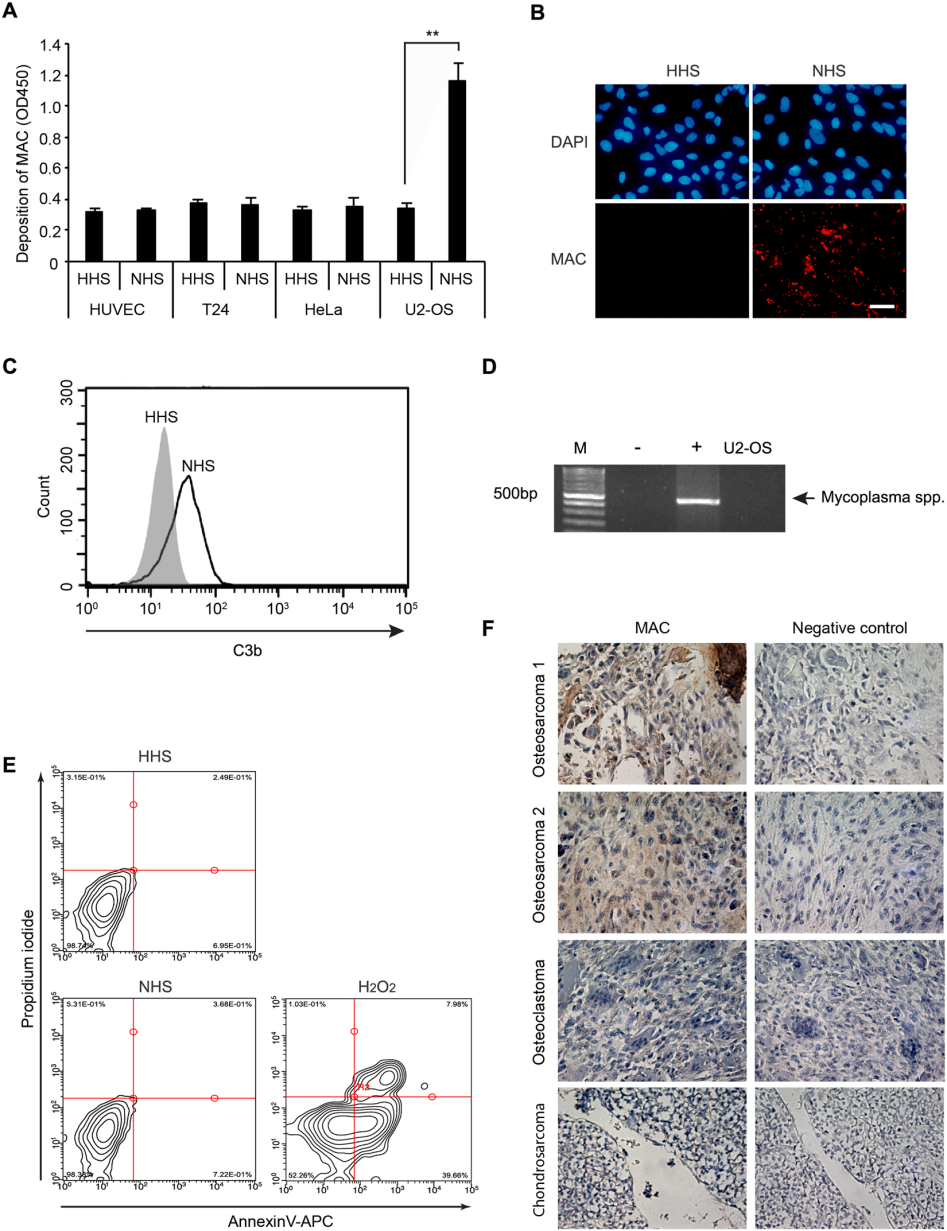
Activation of the complement system in an osteosarcoma cell line. (**A**) Screening of membrane attack complex (MAC) deposition on human cells using a cell-based enzyme-linked immunosorbent assay (ELISA). Cultured cells on 96 well plates were treated with heat-inactivated human serum (HHS) or normal human serum (NHS) for 30 min before cell-based ELISA for C5b-9. Results are shown as mean ± SD, N = 3, **p < 0.01. (**B**) MAC deposition on human bone osteosarcoma epithelial cells (U2-OS). Cells were treated with NHS for 30 min, followed by analysis of MAC deposition by immunofluorescence assay (IFA). Nuclei were stained with DAPI. C5b-9: deposition of C5b-9, detected with anti-C5b-9 antibody. Scale bar = 50 μm. (**C**) Surface deposition of C3b was analyzed by flow cytometry. NHS- or HHS-treated U2-OS cells were analyzed using anti-C3b antibody. Gray and white areas represent the isotype control and anti-C3b antibody, respectively. (**D**) Detection of mycoplasma contamination by PCR with specific primers. M: size marker; -: negative control;+: positive control; U2-OS: PCR reaction with the supernatant from a confluent culture of U2-OS cells. Full-length gels are included in a Supplementary Information file. (**E**) Apoptosis analysis of HHS- or NHS-treated U2-OS cells by flow cytometry with Annexin V-APC/PI staining. PI, Propidium Iodide. (**F**) Detection of MAC deposition on human osteosarcoma. Representative illustration of immunohistochemical detection of MAC deposition (magnification, 400x). Negative control staining was conducted with rabbit normal serum instead of anti-C5b-9 antibody.

Previously, we established the cell-based enzyme-linked immunosorbent assay (ELISA) technique to quantify the complement activation in eukaryotic cell surface^13^. With this method, we screened some cell lines for complement activation. Interestingly, the osteosarcoma cell line, U2-OS, activated the complement system through the addition of NHS (Fig. 1A). To confirm if U2-OS cells can activate the complement system, the deposition of MAC and C3b on cells were analyzed by an immunofluorescence assay (IFA) and flow cytometry, respectively (Fig. 1B,C). To exclude the possibility of complement activation by mycoplasma contamination, detection of mycoplasma was tested by PCR and the results indicated no contamination (Fig. 1D). After complement activation, cell viability was analyzed. Only few apoptosis and cell death was observed both in NHS- and HHS-treated cells (Fig. 1E), suggesting that the activated complement system does not induce cell death in U2-OS cells. These results indicate that U2-OS cells have a potential to be used for complement activation with sublytic level of MAC. To investigate the deposition of MAC on osteosarcoma human tissue, bone and cartilage cancer tissue microarray slide was stained with the anti-MAC antibody (Fig. 1F). In osteosarcoma tissues, obvious staining of MAC was observed on the tumor cells. A non-immune rabbit serum, which was used as a negative control, did not induce any positive signal in the osteosarcoma lesions. Very weak or no MAC staining was observed in the osteoclastoma and chondrosarcoma tissues in the same microarray slide, suggesting MAC does not deposit on all kinds of cancer cells in bone or cartilage. Detection of MAC deposition on the osteosarcoma tissues indicates the complement system is activated in human osteosarcoma.

### Alternative pathway of complement system was activated in U2-OS cells

**Figure 2:**
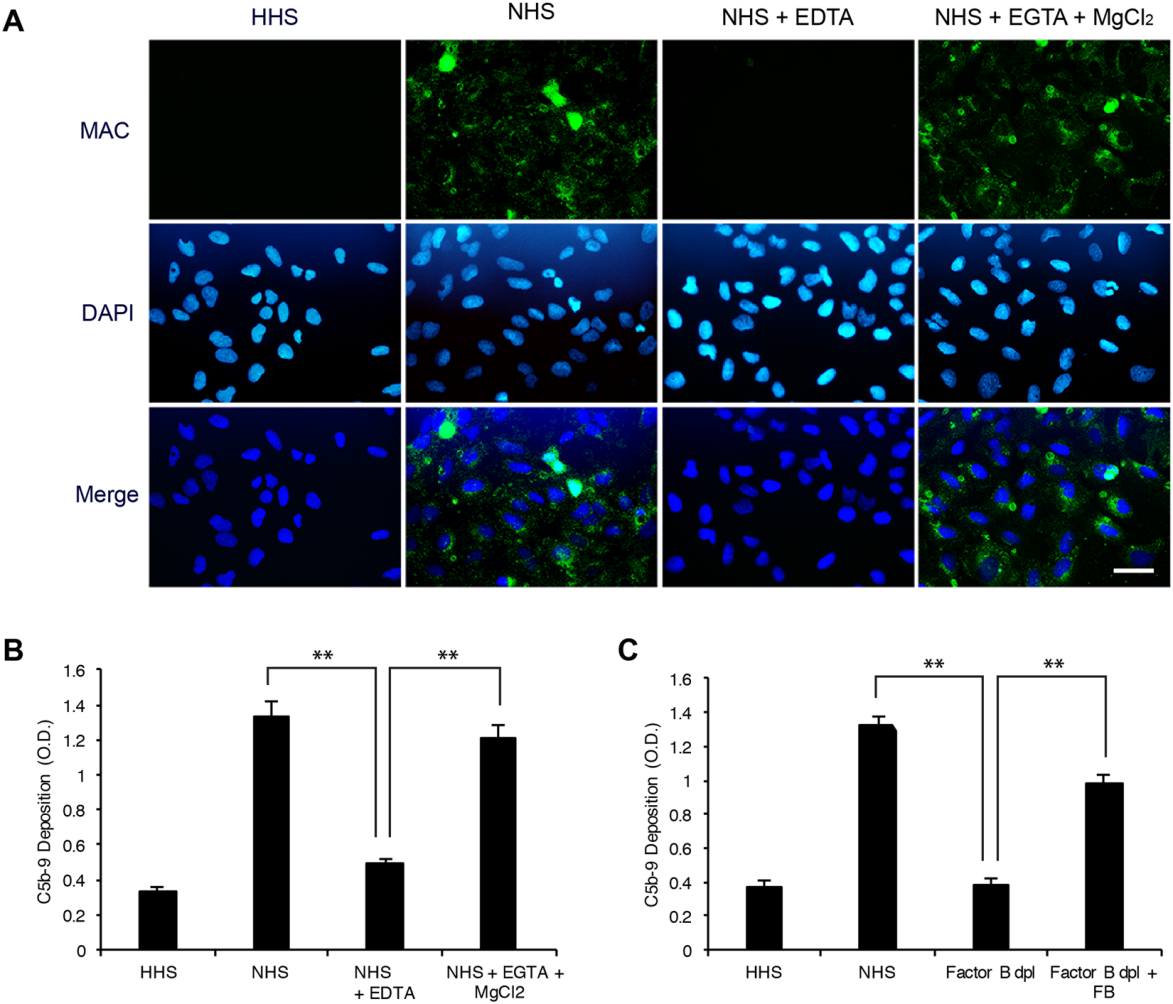
Alternative pathway was activated in U2-OS cells. (**A**,**B**) EDTA but not EGTA and MgCl_2_ abolish complement activation in U2-OS cells. Cells incubated with NHS or NHS containing 20 mM EDTA or 10 mM EGTA with 20 mM MgCl_2_ were examined for C5b-9 deposition by IFA and cell-ELISA. Scale bar: 50 μm. (**C**) Factor B is required for complement activation in U2-OS cells. Cells treated with HHS, NHS, factor B-depleted human serum (factor B dep), and factor B-depleted human serum compensated with factor B were examined for C5b-9 deposition by cell-ELISA. Results are shown as mean ± SD, N = 3, **p < 0.01.

To investigate which pathway of the complement system was involved in U2-OS cells, we preincubated the cells with NHS containing 10 mM ethylenediaminetetraacetic acid (EDTA), which inhibits the activation of all complement pathways or 10 mM ethyleneglycotetraacetic acid (EGTA) with 2 mM MgCl_2_, which inhibit the antibody-dependent classical pathway. While EDTA-treated cells no longer had C5b-9 deposition, cells treated with EGTA together with MgCl_2_ continue to have C5b-9 deposition (Fig. 2A,B), indicating that C5b-9 deposition on U2-OS cells was mediated by the complement pathway, most likely through the alternative complement pathway. To confirm whether alternative complement pathway was activated, we examined C5b-9 deposition after depleting factor B from NHS. As expected, human serum with depletion of factor B failed to induce C5b-9 deposition; however, addition of factor B to the depleted human serum rescued the C5b-9 deposition (Fig. 2C). Together, the above results indicated that the alternative complement pathway was activated in U2-OS cell.

### Negative regulatory proteins of the complement system were suppressed in U2-OS cells

**Figure 3:**
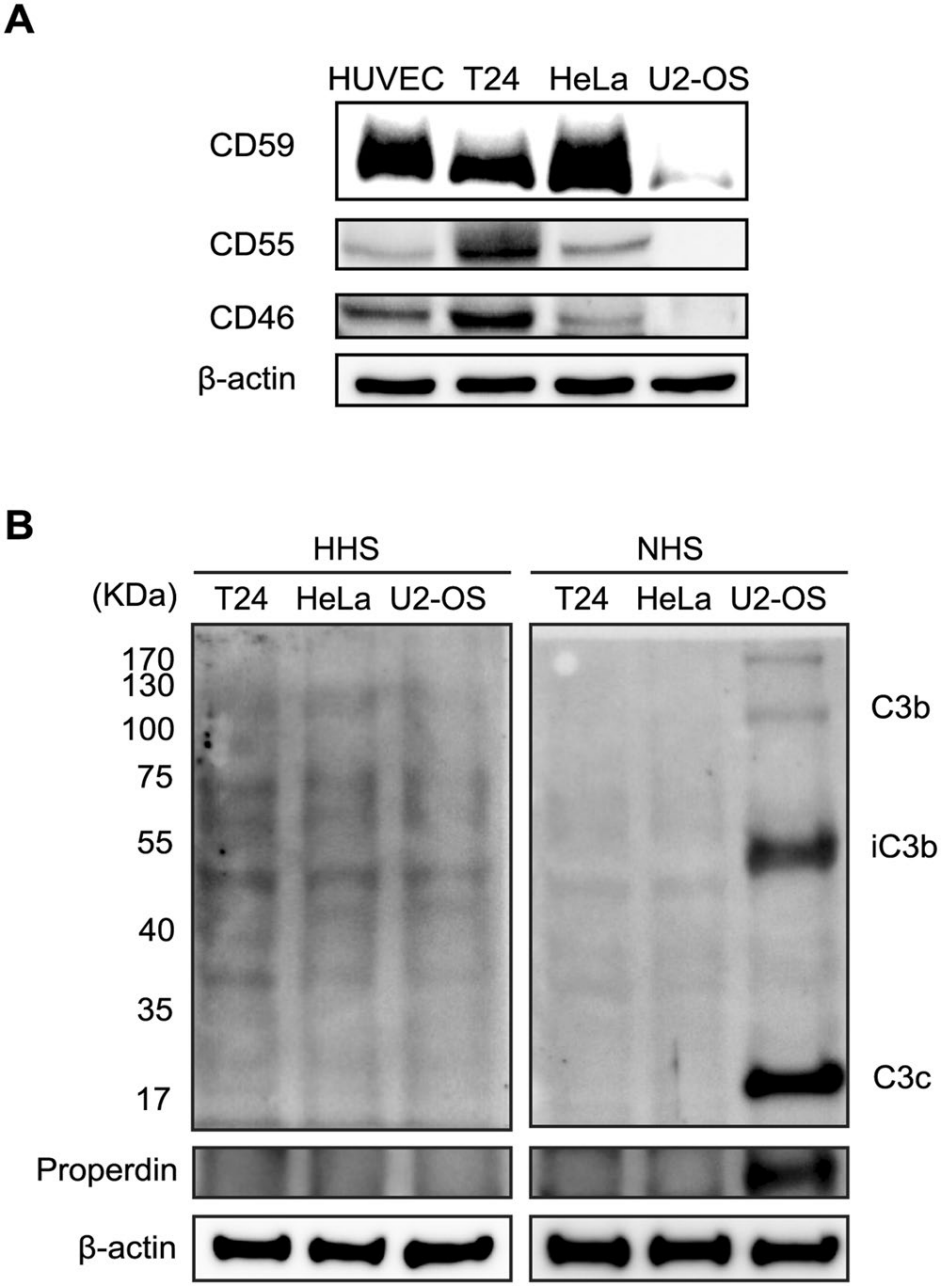
Negative regulatory proteins of the complement system were suppressed in U2OS cells. (**A**) Analysis of the expression of CD46, CD55, and CD59 in human endothelial cells and human cancer cells by Western-blotting. The grouping of blots cropped from different gels and full-length blots are included in a Supplementary Information file. (**B**) Analysis of C3 and properdin in human cancer cells by Western blotting. β-actin was used as a loading control and the full-length blot for β-actin are included in a Supplementary Information file.

There are various mechanisms controlling the activation of the complement system. Some complement regulatory proteins including CD46, CD55, and CD59 on cell surfaces inhibit complement activation. We investigated whether the expression of these proteins has correlation with complement activation. Interestingly, the expression of CD46, CD55, and CD59 was suppressed in U2-OS cells compared with human endothelial cells or other cancer cells which did not activate the complement system (Fig. 3A). Endogenous expression of properdin or C3 is also related with complement activation^14^, we examined their expression on U2-OS cells. Because C3b is deposited on the cell surface by complement activation and properdin is incorporated into C3 convertase during activation of the alternative pathways, NHS-treated U2-OS cells were used as a positive control. Except positive control, no evidence for the endogenous expression of C3 or properdin was observed in U2-OS cells (Fig. 3B). Together, activation of the complement system on U2-OS cells would be mediated by suppression of the negative regulatory proteins of the complement system. Recent studies suggested that microRNA expression would be associated with the expression of complement regulatory proteins^15,16^. Therefore, we analyzed the previously reported microRNAs related with CD46 and CD55 in U2-OS cells. Interestingly, most analyzed microRNAs were significantly upregulated in U2-OS cells compared to other cancer cell lines, suggesting microRNAs might be one of the mechanisms for the regulation of these proteins (Fig. S1).

### Complement activation of U2-OS cells increased angiogenesis of human endothelial cell through secreted factors

**Figure 4:**
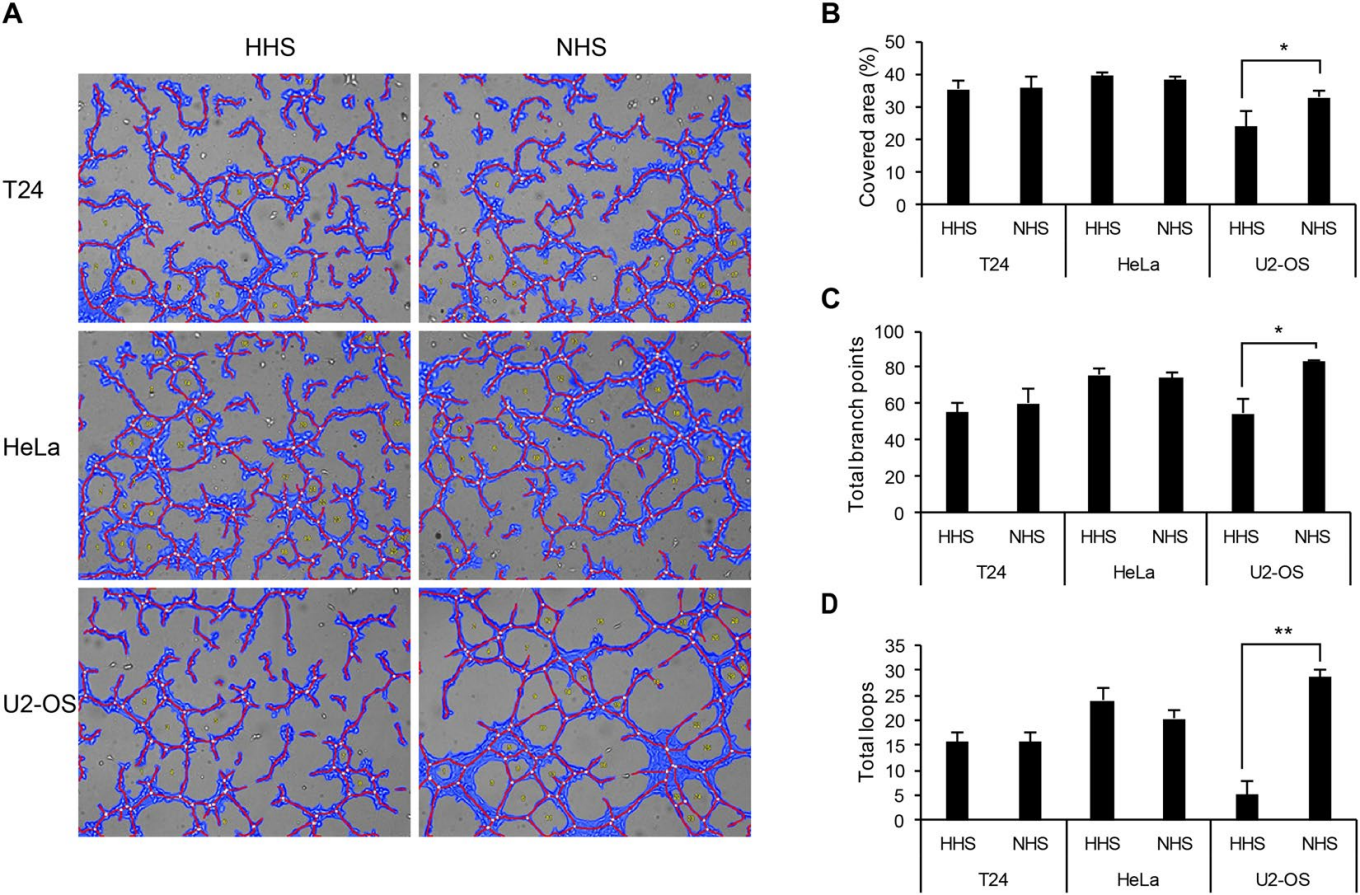
Activation of the complement system in U2-OS cells enhanced *in vitro* tube formation. (**A**) Representative pictures for angiogenic activity of human endothelial cells treated with the supernatant from cancer cells. Cells were treated with either NHS or HHS followed by their supernatants and then HUVEC addition on Matrigel. After incubation for 1–6 h, random microscopic fields (magnification, x100) were photographed and analyzed using Wimtube software. (**B**–**D**) Covered area, total branch points, and total loops were analyzed per field and results from random three fields were shown as mean ± SD, N = 3, *p < 0.05, **p < 0.01.

To investigate if complement activation affects angiogenic activity in endothelial cells through the secretion of specific factors, cancer cells were treated with NHS or HHS for 1 h followed by changing culture media without human serum conditioned media. After 48 h, both conditioned media were collected and applied to human endothelial cells. While the supernatant from NHS-treated U2-OS cells enhanced *in vitro* tube formation of human endothelial cells, conditioned media from HeLa and T24 cells did not increase angiogenic effects (Fig. 4), suggesting that complement activation of U2-OS is associated with enhanced production of angiogenesis-related secreted factors.

### Increased production of VEGF-A and FGF1 in complement activated U2-OS cells

**Figure 5:**
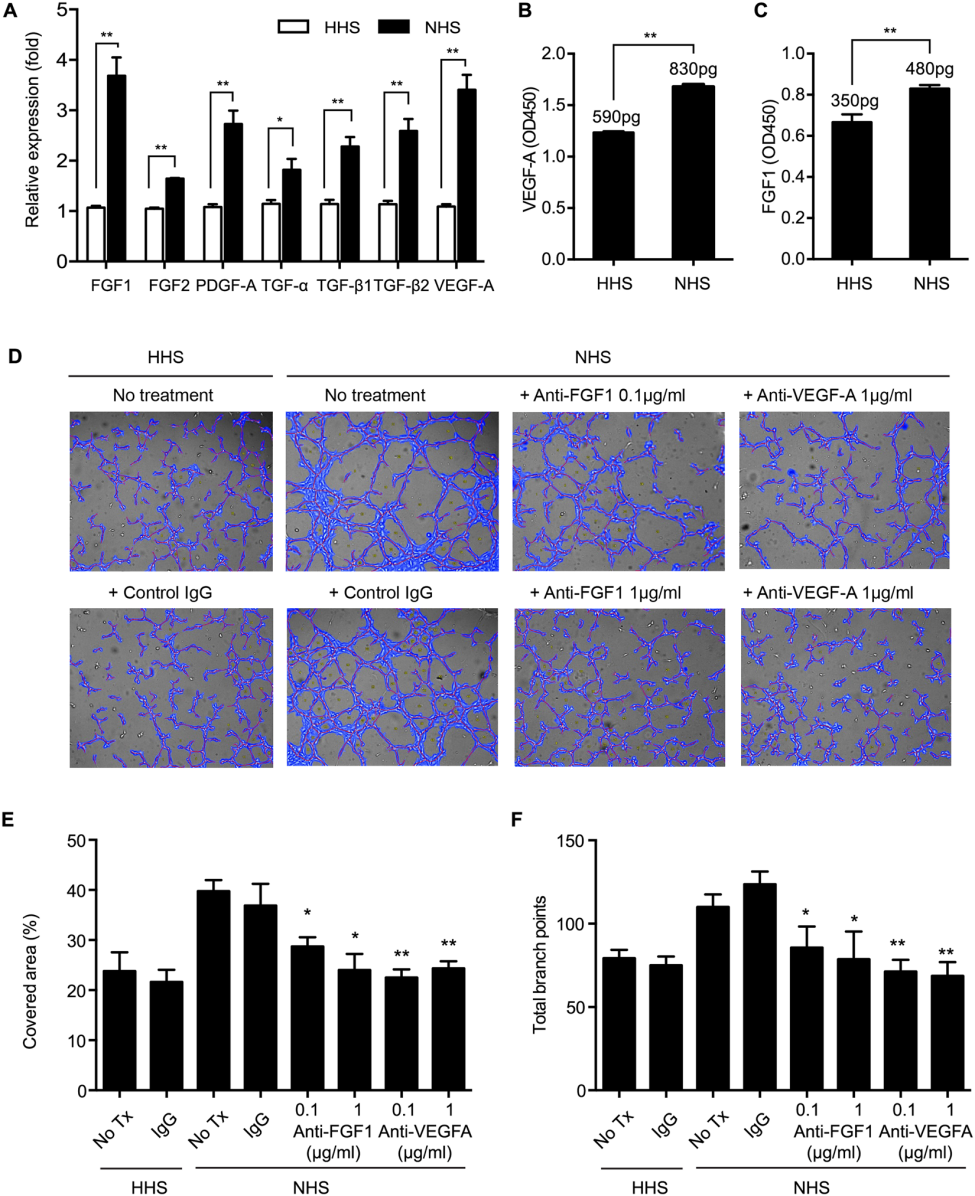
Complement activation of U2-OS cells enhanced expression of angiogenesis-related growth factors. (**A**) Quantitative RT-PCR for angiogenesis-related growth factors in HHS- or NHS-treated U2-OS cells. mRNA expression levels for each gene were normalized to the GAPDH expression level. These results represent the mean of triplicate experiments. Standard deviations are indicated by error bars. *p < 0.05, **p < 0.01 (**B**,**C**) Quantities of VEGF-A (**B**) and FGF1 (**C**) secreted from the NHS- or HHS-treated U2-OS cells were determined by ELISA. Results are shown as mean ± SD, N = 3, **p < 0.01. (**D**–**F**) Neutralizing effects of anti-FGF1 and anti-VEGF-A antibody for angiogenic activity of human endothelial cells treated with the supernatant from U2-OS cells. The representative microscopic fields (magnification, x100) were analyzed by Wimtube software (**D**). Covered area (**E**) and total branch points (**F**) were analyzed from three random fields of each experimental condition. Data shown as mean ± SD, N = 3, *p < 0.05, **p < 0.01.

To determine which secreted factors from NHS-treated U2-OS cells enhance angiogenesis *in vitro*, various angiogenesis-related growth factors were analyzed by RT-qPCR (Fig. 5A). mRNAs of several growth factors were upregulated in NHS-treated U2-OS cells as compared to HHS-treated cells. Since VEGF-A and FGF1 showed the most significant difference between NHS- and HHS-treated cells, these growth factors were quantified by ELISA using the supernatant from NHS- or HHS-treated U2-OS cells. VEGF-A and FGF1 expression in complement activated cells was significantly higher than in HHS-treated cells (Fig. 5B,C). To confirm the association of the production of VEGF-A/FGF1 and angiogenesis, neutralizing antibodies against them were applied to *in vitro* tube formation assay. As expected, both antibodies significantly suppressed tube formation by the supernatant from NHS-treated cells (Fig. 5D–F).

### The increase of *in vitro* angiogenesis in U2-OS cells is regulated by the phospho-ERK signaling pathway

**Figure 6:**
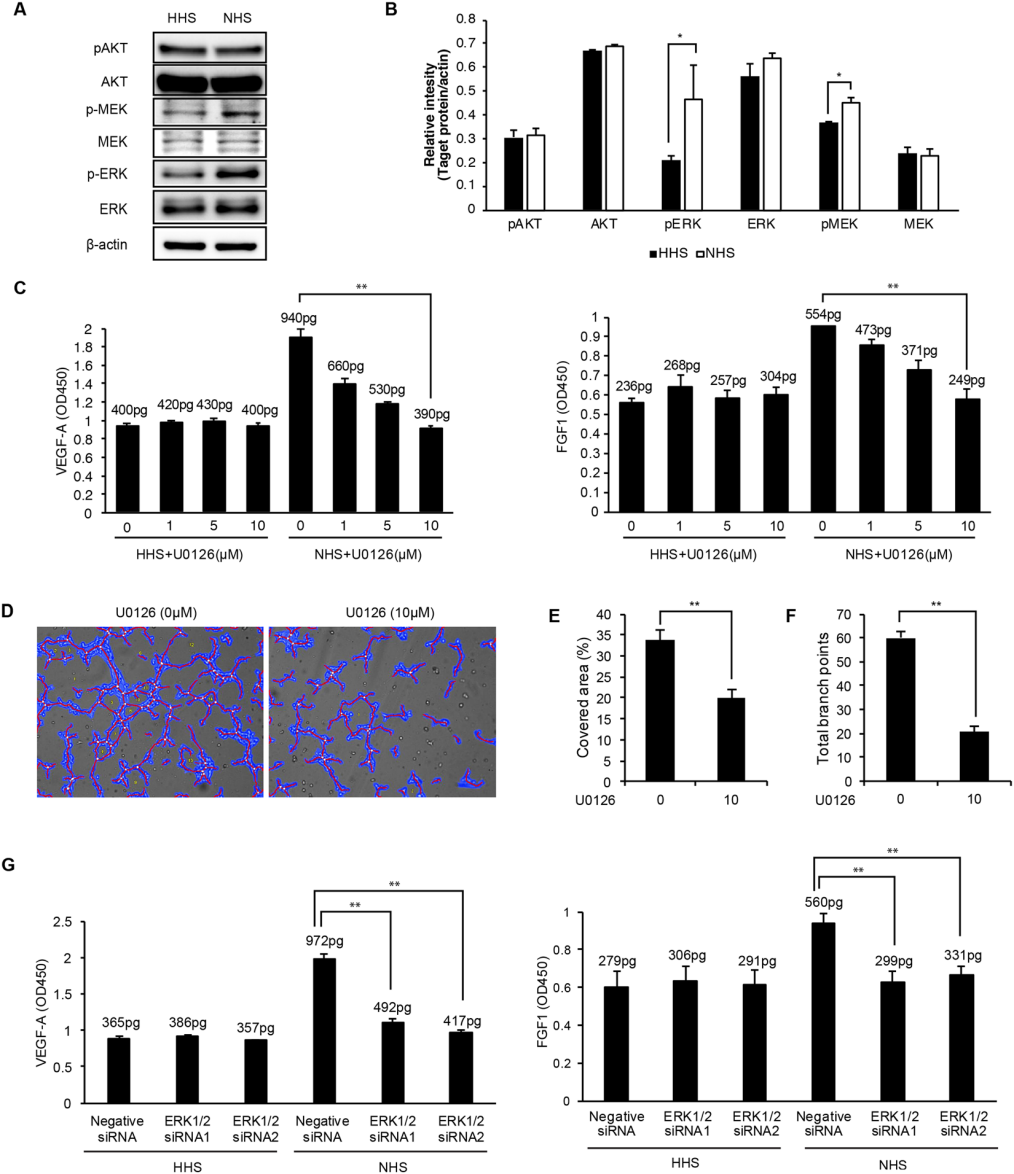
Complement-mediated production of angiogenic factors was associated with ERK phosphorylation. (**A**) ERK, phosphor-ERK(pERK, Thr202), MEK, phosphor-MEK, AKT, and phosphor-AKT(pAKT, Thr308) were analyzed by western blotting. β-actin was used as a housekeeping gene. The grouping of blots cropped from different gels and full-length blots are included in a Supplementary Information file. (**B**) Densitometric analysis for pAKT, pMEK, and pERK from three independent western blot analyses. (**C**) Inhibition of ERK pathway suppressed VEGF-A and FGF1 production in HHS- or NHS-treated U2-OS cells. U2-OS cells were treated with NHS for 30 min with varying concentrations of U0126 inhibitor. The culture media was replaced with fresh media including U0126. After 48 h, VEGF-A or FGF1 in the supernatant was analyzed by ELISA. (**D**–**F**) Inhibition of ERK pathway suppressed the angiogenic activity of the supernatants from NHS-treated U2-OS cells. The supernatants from NHS-treated U2-OS with or without U0126 were collected, and they were added with HUVECs on Matrigel. The representative microscopic fields (magnification, x100) were analyzed by Wimtube software (**D**). Covered area (**E**) and total branch points (**F**) were analyzed from three random fields of each experimental condition. (**G**) Knockdown of ERK by siRNA suppressed the production of VEGF-A and FGF in NHS-treated U2-OS cells. Data shown as mean ± SD, N = 3, **p < 0.01.

The ERK1/2 and AKT pathways are known to be activated through complement activation by sublytic MAC or C3a^17^. Both signaling pathways are also implicated in angiogenesis and the production of VEGF/FGF1^18–20^. Therefore, we investigated if the AKT or ERK pathways are activated in NHS-treated U2-OS cells. Interestingly, the phosphorylation of ERK was higher in NHS-treated U2-OS cells compared to HHS-treated U2-OS cells, which may represent a mechanism for the enhanced angiogenesis of complement activated U2-OS cells (Fig. 6A,B). To investigate the association between VEGF-A/FGF1 production and phosphorylation of ERK, U0126 inhibitor was applied to HHS- or NHS-treated U2-OS cells and the supernatant was isolated after 48 h of incubation with varying concentrations of U0126. Western blot analysis of the cell lysates and ELISA for each supernatant showed that the phosphorylation of ERK and VEGF-A/FGF1 decreased in a U0126 dose-dependent manner, respectively (Figs S2 and 6C). Additionally, angiogenic activity of the supernatant from NHS-treated U2-OS cells was analyzed to find if there is a link between U0126 suppressed growth factors and *in vitro* tube formation activity. The analysis showed that angiogenic activity was significantly decreased by treatment of U0126 (Fig. 6D–F). To confirm the association of ERK and induction of VEGF-A/FGF1 by the complement system, ERK-1/2 was suppressed by siRNAs (Fig. S3). Knockdown of ERK-1/2 with siRNAs also significantly suppressed the expression of VEGF-A/FGF1 in NHS-treated U2-OS cells (Fig. 6G). Together, our results suggest that the production of angiogenesis-related growth factors (VEGF-A and FGF1) in U2-OS cells through complement activation is mediated by ERK signaling pathway.

## Discussion

Although there is increasing research in uncovering the biological responses of the complement system, its association with cancer progression remains controversial. Complement activation is considered damaging to cancer cells through complement-dependent cytotoxicity, which is recruited by anti-tumor monoclonal antibodies^21^. On the other hand, several studies have demonstrated the pro-tumor effects of the complement system in different experimental conditions^3^. Since the complement system has diverse roles depending on the microenvironment, it is not easy to elucidate the exact role of the complement system *in vivo*. Previous studies have suggested a role for complement proteins in angiogenesis, whereby C3a and C5a stimulate the secretion of VEGF in adjacent retinal pigmented epithelium and choroid cells in a dose-dependent manner^22^. C3 and MAC are deposited in laser-induced choroidal neovascularization, subsequently causing increases in VEGF and other angiogenic growth factors^23^. Using a novel *in vitro* model with sublytic levels of complement activation in cancer cells, we demonstrated that complement activation in cancer cells can lead to increased production of angiogenic growth factors. Furthermore, phosphorylation of ERK is associated with the complement-mediated production of VEGF-A and FGF1.

Complement activation is associated with cell death through MAC; however, in our previous and current studies, we have not seen any evidence for cell death of human endothelial cells, human mesenchymal stem cells, and various cancer cells through MAC deposition^13,24,25^. A possible explanation for the reason is that our *in vitro* model of complement activation was not mediated by antigen-antibody complex but through alternative pathway, which would only induce sublytic levels of C5b-9 deposition.

When NHS was applied to cancer cells, complement activation was observed in some cancer cell lines^13^. We found that the expression of complement inhibitory proteins was suppressed in U2-OS cells, which could be a reason for complement activation in eukaryotic cells^24,26,27^. A recent study suggested that microparticles released from cells cause activation of the alternative pathway of the complement system^28^.

Increasing evidence supports the nonimmunological function of the complement system, in which complement activation in the tumor microenvironment enhances tumor growth and increases metastasis^2,3^. MAC can upregulate oncogenic growth factors and cytokines to sustain tumorigenesis and angiogenesis, whose autocrine and paracrine actions promote tumor invasiveness and metastasis^2^. Anaphylatoxins, C3a and C5a, mediated by the activation of M2 macrophages, can also regulate angiogenesis^29^. However, more research is needed to elucidate the exact effects and mechanisms of the sublytic level of complement activation on cancer.

In our present study, we demonstrated the activation of the complement system in an osteosarcoma cell line, U2-OS, through NHS treatment. This activation enhanced angiogenic activity through the secretion of growth factors. Since the relationship between the complement system and tumors remains unclear, a complete theoretical framework has not emerged. This study presents a direct linkage of the complement system and angiogenesis in an *in vitro* cancer cell model, which could be useful in elucidating the relationship between the complement system and tumors and the underlying mechanisms.

## Methods

### Cell culture and reagents

U2-OS, HeLa, and T24 cells were obtained from the Korean Cell Line Bank (Seoul, South Korea). The cells were cultured in Dulbecco’s modified Eagle’s medium (DMEM; GE Healthcare, Little Chalfont, UK) supplemented with 10% fetal bovine serum (FBS; Welgene, Seoul, South Korea) and 1% antibiotics (Lonza, Allendale, NJ). Human umbilical vein endothelial cells (HUVECs) were purchased from Lonza and cultured in endothelial cell growth medium-2 (EGM-2; Lonza) bullet kit. The cells were maintained in a humidified atmosphere of 5% CO_2_ at 37 °C. Pooled complement human serum was purchased from Innovative Research, Inc (Novi, MI) and used as normal human serum (NHS) in all experiments. Heat-inactivation was performed using this serum at 56 °C for 30 min and used as heat-inactivated human serum (HHS).

### C5b-9 cell-ELISA

The cell-ELISA was performed as described with modifications^13^. Briefly, Cells were seeded at 10,000 cells/well in 96-well culture plates and incubated overnight at 37 °C in 5% CO_2_. Cells were then cultured in media containing 10% pooled human serum (Innovative Research, Novi, MI) for 1 h to activate the complement system. Plates were washed with phosphate-buffered saline (PBS) followed by fixing with 3% paraformaldehyde (PFA) for 15 min. The cells were incubated in blocking buffer (5% skim milk in Tris-buffered saline; TBS) for 1 h at 37 °C. A rabbit polyclonal C5b-9 antibody (Abcam, Cambridge, MA) diluted in blocking buffer (1:4,000) was added to the plate, and incubated with the cells for 2 h. The plate was washed three times with TBS/T for 15 min, and horseradish peroxidase (HRP)-conjugated anti-rabbit IgG (GE Health Care, Buckinghamshire, UK) was added. After incubation at room temperature for 1 h, the substrate 3,3′,5,5′-tetramethylbenzidine (TMB, KPL, Gaithersburg, MD) was used. The absorbance at 450 nm was measured by a microplate reader (Molecular Devices, Silicon Valley, CA).

### Immunofluorescence assay

Cells were seeded onto microscope cover glass. After culturing overnight, culture media containing 10% pooled human serum or heat-inactivated human serum was treated for 1 h. The cells were fixed with 4% PFA in PBS and blocked with 3% bovine serum albumin. Cells were incubated with rabbit polyclonal anti-C5b-9 (1:500, Abcam) overnight at 4 °C, followed by incubating with Alexa Fluor 588-labeled secondary antibodies (Invitrogen, Carlsbad, CA). After washing, nuclei were stained using 4,6-diamidino-2-phenylindole and mounted in Vectashield® (Vector Laboratories Inc., Burlingame, CA). Images were observed under the Nikon Eclipse E400 microscope (Nikon Instruments Inc., Melville, NY) using Nikon Digital site DS-U2, and analyzed using NIS element F.

### Flow cytometry

For the detection of C3b, cells were treated with culture media containing 10% pooled human serum before trypsinization. The cells were incubated with mouse monoclonal anti-C3b antibody (Thermo Scientific, Rockford, IL) or control IgG (Santa Cruz, Santa Cruz, CA) for 30 min on ice. After washing, allophycocyanin (APC)-conjugated goat anti-mouse IgG (R&D systems, Minneapolis, MN) was added for 30 min at 4 °C. After washes, cells suspended in 1% FBS/PBS were analyzed using a Guava easyCyte Flow Cytometer and the InCyte 3.1 software (Merck Millipore, Bedford, MA). For apoptosis analysis, eBioscience Annexin V apoptosis detection kit APC (Thermo Scientific) was used as manufacturer’s instructions.

### *In vitro* endothelial cell tube formation assay

Matrigel (BD biosciences, San Jose, CA) was coated on μ-slide Angiogenesis plates (ibidi, GmbH, Germany). HUVECs (10,000 cells/well) were placed on prepared Matrigel matrix with culture media. The plate was incubated at 37 °C with 5% CO_2_ and angiogenic activity was analyzed in three random fields of wells using the WimTube software (Onimagin Technologies SCA, Cordoba, Spain)^30^.

### Quantitative real-time reverse transcription PCR (RT-qPCR)

Total RNA from cells was isolated by NucleoSpin RNA II as recommended by the manufacturer (MACHEREY-NAGEL Inc., Bethlehem, PA). Total RNA was reverse-transcribed to obtain the first-strand cDNA using the ReverTra Ace qPCR RT kit (TOYOBO CO, Osaka, Japan). Real-time PCR was performed using the SYBR^®^ FAST qPCR mix (Takara, Otsu, Japan). The cycling conditions were as follows: 95 °C for 30 sec, 40 cycles of 95 °C for 5 sec, and 60 °C for 10 sec. Specificity of the amplified products was confirmed by analyzing the melting curves. All samples were tested in triplicate using glyceraldehyde 3-phosphate dehydrogenase (GAPDH) as a control. The primers were synthesized by GENOTECH (Daejeon, South Korea) and the following primers were used: FGF1s; 5′-TTATACGGCTCACAGACA-3′ and FGF1as: 5′-ATAGGTGTTGTAATGGTTCTC-3′ for human FGF1; FGF2s: 5′-GAGAGGAGTTGTGTCTATCA-3′ and FGF2as: 5′-GCCAGTAATCTTCCATCTTC-3′ for human FGF2; VEGFAs: 5′-CACGAAGTGGTGAAGTTC-3′ and VEGFAas: 5′-AGGATGGCTTGAAGATGT-3′ for human VEGFA; PDGFA S: 5′-CAGTCAGATCCACAGCAT-3′ and PDGFA as: 5′-TTCTCTTCCTCCGAATGG-3′ for human PDGFA; TGFαs: 5′-CTGTTCGCTCTGGGTATT-3′ and TGFαas: 5′-ACTGAGTGTGGGAATCTG-3′ for human TGFα; TGFβ1s: 5′-CTATGACAAGTTCAAGCAGAG-3′ and TGFβ1as: 5′-GAGGTATCGCCAGGAATT-3′ for human TGFβ1; TGFβ2s: 5′-GTGAAGAACTAGAAGCAAGATT-3′ and TGFβ2as: 5′-GCAATAACATTAGCAGGAGAT-3′ for human TGFβ2; GAPDHs: 5′-GGTATCGTGGAAGGACTC-3′ and GAPDHas: 5′-GTAGAGGCAGGGATGATG-3′ for human GAPDH.

As for the miRNA analysis, total RNA was extracted using TRIzol Reagent (Invitrogen). The cDNAs were synthesized by Mir-X miRNA qRT-PCR SYBR kit (Clontech Laboratories, Palo Alto, CA) according to the manufacturer’s guidelines. Each miRNA sequence was regarded as miRNA-specific 5′ primers and the mRQ3′ primers supplied by the kit were used as the 3′ primers. All reactions, including no template controls, were run triplicate. miRNA expression in cells is normalized to U6 snRNA. The sequence of primer was: 5′-AGTTTTGCATAGTTGCACTACA-3′ for miR19a; 5′-TAAAGTGCTTATAGTGCAGGTAG-3′ for miR-20a; 5′-TGGAGAGAAAGGCAGTTCCTGA-3′ for miR-185; 5′-ATATAATACAACCTGCTAAGTG-3′ for miR-374b; 5′-CATCTTACTGGGCAGCATTGGA-3′ for miR-200b; 5′-CGTCTTACCCAGCAGTGTTTGG-3′ for miR-200c; 5′-TACTGCATCAGGAACTGATTGGA-3′ for miR-217; 5′-AACGCTTCACGAATTTGCGT-3′ and 5′-CTCGCTTCGGCAGCACA-3′ for U6.

### VEGF-A and FGF1 ELISA

A total of 1 × 10^6^ cells was seeded in 75 cm^2^ cell culture flasks with DMEM containing 10% FBS. To activate the complement system, the culture media was replaced by DMEM containing 10% pooled normal human serum (NHS) and the cells were incubated for 1 h. After complement activation, cells were washed with PBS twice. Then the media was replaced again with FBS-free DMEM. The conditioned medium was collected after 48 h of incubation. Concentrations of VEGF-A or FGF1 in conditioned medium were measured by using the human VEGF-A or FGF1 ELISA kit (Elabscience, Houston, TX) according to the manufacturer’s instructions.

### Western blotting

Western blotting was performed as previously described with modifications^24^. Cell proteins were isolated using 1 × RIPA buffer with protease inhibitor and phosphatase inhibitor. The proteins were resolved by electrophoresis in a 12% SDS-polyacrylamide gel and transferred onto a nitrocellulose membrane (GE Healthcare). The membranes were blocked with 5% skim milk in Tris-buffered saline with 0.1% Tween 20. Mouse monoclonal anti-C3b (Thermo scientific), mouse monoclonal anti-properdin (Abcam), rabbit polyclonal anti-CD55 (Santa Cruz biotechnolofy), rabbit polyclonal anti-CD59 (Abcam), rabbit polyclonal anti-CD46 (Santa Cruz Biotechnology), mouse monoclonal anti-ERK(1/2) (Bioss Antibodies Inc., Woburn, MA), rabbit polyclonal anti-phospho ERK (Bioss Antibodies Inc.), rabbit polyclonal anti-AKT (Bioss Antibodies Inc.), rabbit polyclonal anti-phospho AKT (Cell Signaling Technology, Beverly, CA) and mouse monoclonal anti-β-actin antibodies (Sigma, St. Louis, MO) were used as primary antibodies. HRP-conjugated anti-rabbit or anti-mouse antibodies (Bethyl Laboratories Inc., Montgomery, TX) were used as secondary antibodies. The results were visualized using an ECL detection reagent (Bio-Rad, Hercules, CA).

### Mycoplasma detecting PCR

Mycoplasma in culture media were detected by the previously described mycoplasma detecting PCR^31^, using the following primers: Mycoplasma Universal s; 5′- ACACCATGGGAGCTGGTAAT -3′ and Mycoplasma Universal as; 5′- CTTCWTCGACTTYCAGACCCAAGGCA -3′. The 16 S ribosomal DNA region of the strain with which the cell lines were infected was amplified by PCR. The cycling conditions were as follows: initial denaturation at 95 °C for 2 min, 40 cycles consisting of denaturation at 95 °C for 30 sec, annealing at 58 °C for 30 sec, and extension at 72 °C for 60 sec, followed by a final extension at 72 °C for 5 min. The PCR products were analyzed using 1.5% agarose gel. DNA fragments were visualized with a Gel Doc XR system (Bio-Rad) after being staining with ethidium bromide.

#### Tissue microarray

Human paraffin embedded tissue array slide for bone and cartilage cancer tissue (BO241) was purchased from US Biomax, Inc (Derwood, MD). Tissue sections were deparaffinized in xylene followed by a graded series of alcohol washes prior to staining. Subsequently, all sections were treated with 3% H_2_O_2_ for 10 minutes to block endogenous peroxidase activity, and then slides were blocked with normal goat serum (Vector laboratories, Burlingame, CA) for 1 h at room temperature (RT). After blocking, slides were incubated with the anti-C5b-9 antibody (1:100, Abcam Inc., Cambridge, MA) overnight at 4 °C. Then sections incubated with biotinylated goat anti-rabbit secondary antibodies (1:100, Vector Laboratories, Burlingame, CA) for 1 h at RT, *followed by* 30 min *incubation* with Vectastain avidin-biotin complex reagents (Vectastain-Elite kit, Vector Laboratories, Burlingame, CA). Then color was developed with 3,3′-diaminobenzidine(DAB) and counterstained with Mayer’s hematoxylin. Finally, Slides were observed under an Eclipse E400 microscope (Nikon Instruments Inc., USA) and images were captured with a Nikon Digital Sight DS-U2 camera.

### Statistical analysis

Each experiment is performed at least three times independently, and the representative result has shown. The number of replicates was indicated in each figure legend as “N”. Results are shown as means ± standard deviations. The one-tailed Student’s *t* test was used to assess the significance of difference between groups. Statistical significance at P values of <0.05 and <0.01 is indicated by * and **, respectively.

### Data availability

The dataset analyzed during the current study are available from the corresponding author on reasonable request.

## Electronic supplementary material

Supplementary information
