# Supplementary material for: Activation of the complement system in an osteosarcoma cell line promotes angiogenesis through enhanced production of growth factors
Source: Sci Rep. 2018 Apr 3;8:5415. doi: 10.1038/s41598-018-23851-z (PMC5883033; doi:10.1038/s41598-018-23851-z)
Supplement: Supplementary file 1 — Supplementary information [file 41598_2018_23851_MOESM1_ESM.pdf]

Supplementary Information

Activation of the complement system in an osteosarcoma cell line promotes angiogenesis through enhanced production of growth factors

Hyungtaek Jeon, Seung Ro Han, Suhyuk Lee, Sang june Park, Joo Heon Kim, Seung-Min Yoo, and Myung-Shin Lee

Figure S1.

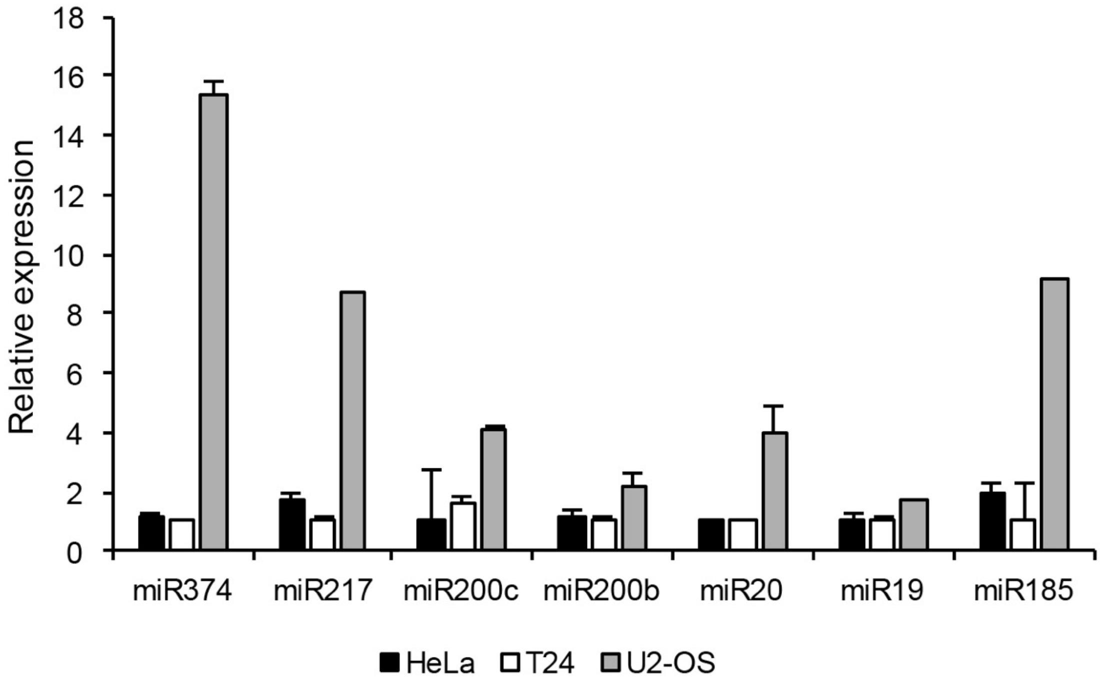

Figure S1. Analysis for the complement regulatory proteins-related microRNA expressions in U2OS, HeLa, and T24. The relative microRNA expression was quantified by referencing upon the U6 expression. Shown is the representative of three independent replicates (N=3).

Figure S2.

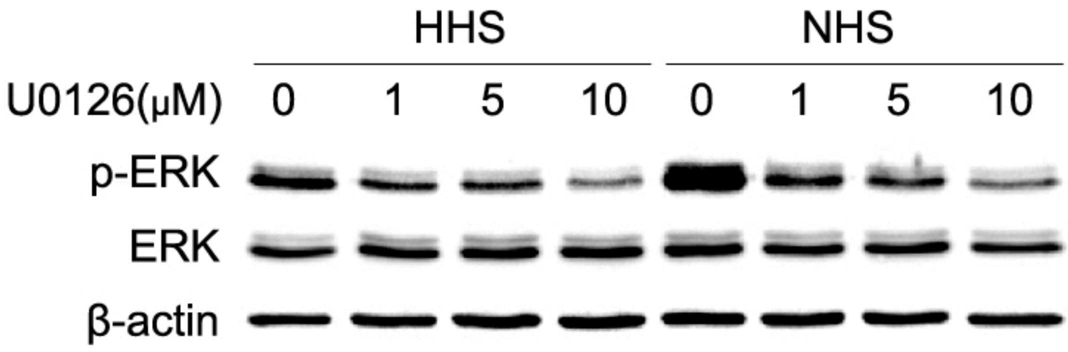

Figure S2. Western blot analysis for various concentration of U0126-treated U2-OS cells. HHS: HHS-treated cells, NHS: NHS-treated cells.

Figure S3.

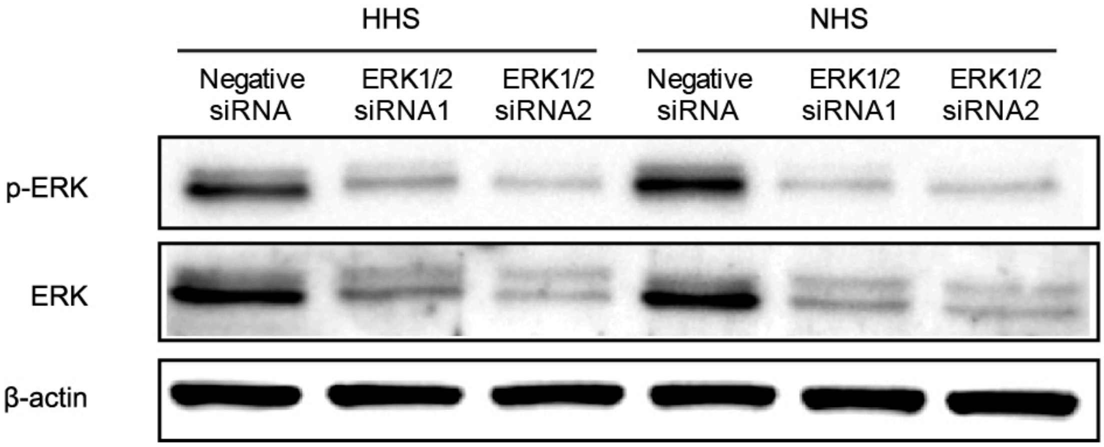

Figure S3. Western blot analysis for siRNA-induced knockdown of ERK.

Figure S4.

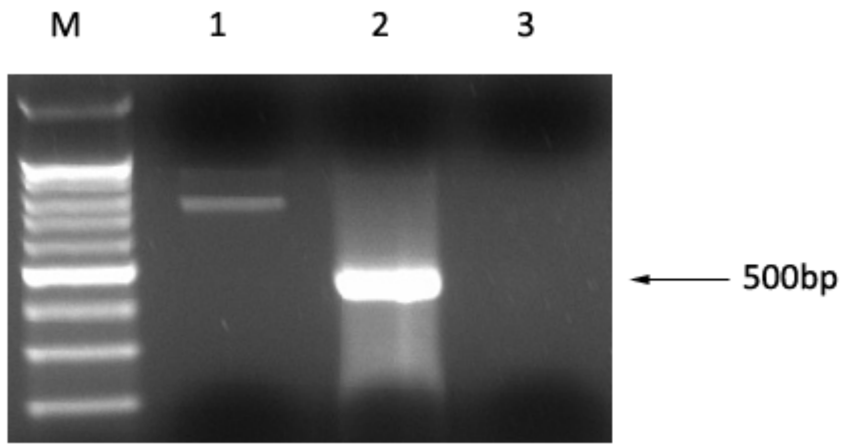

Figure S4. DNA electrophoresis uncut gel data of Figure 1D.

Figure S5.

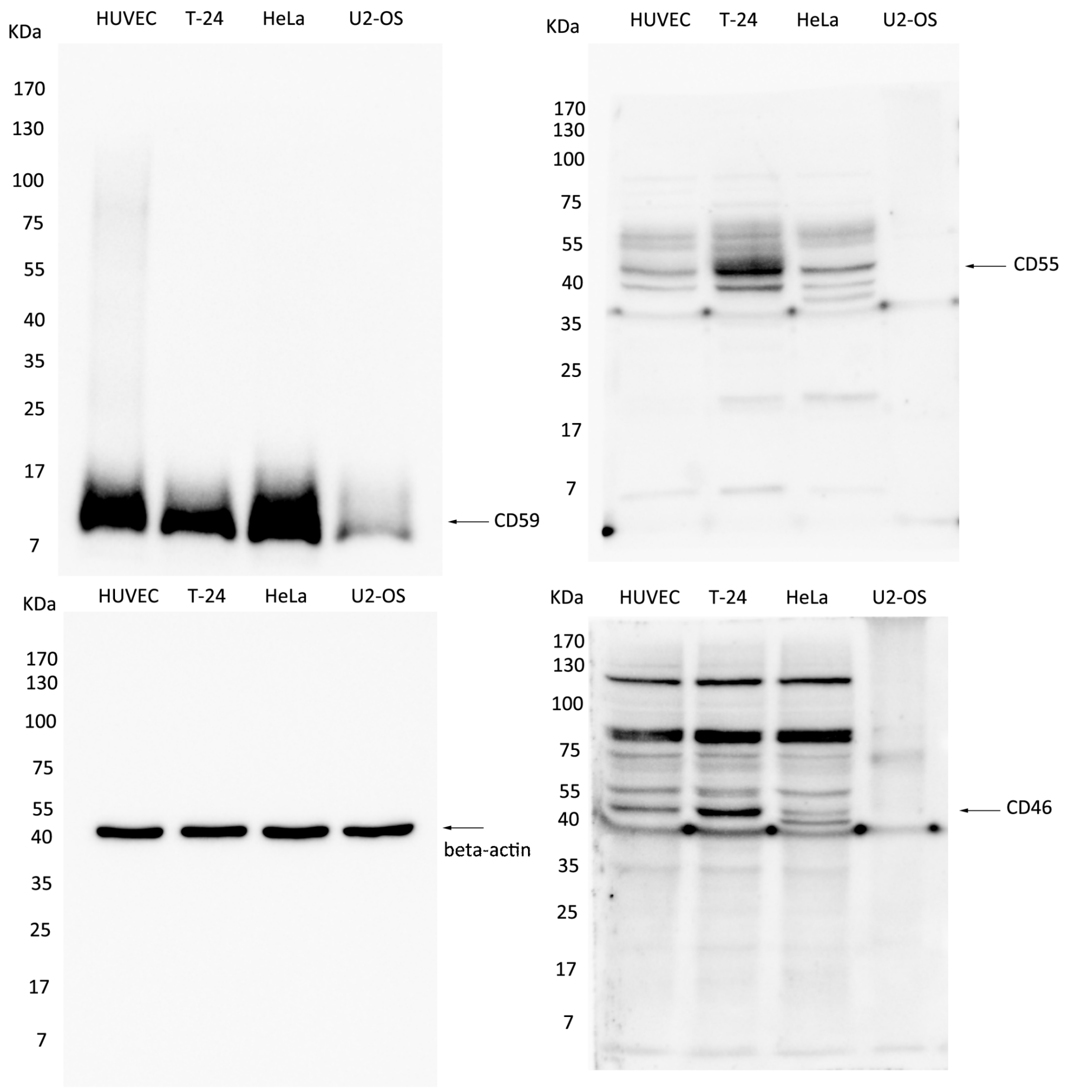

Figure S5. Western blot uncut membrane data of Figure 3A.

Figure S6.

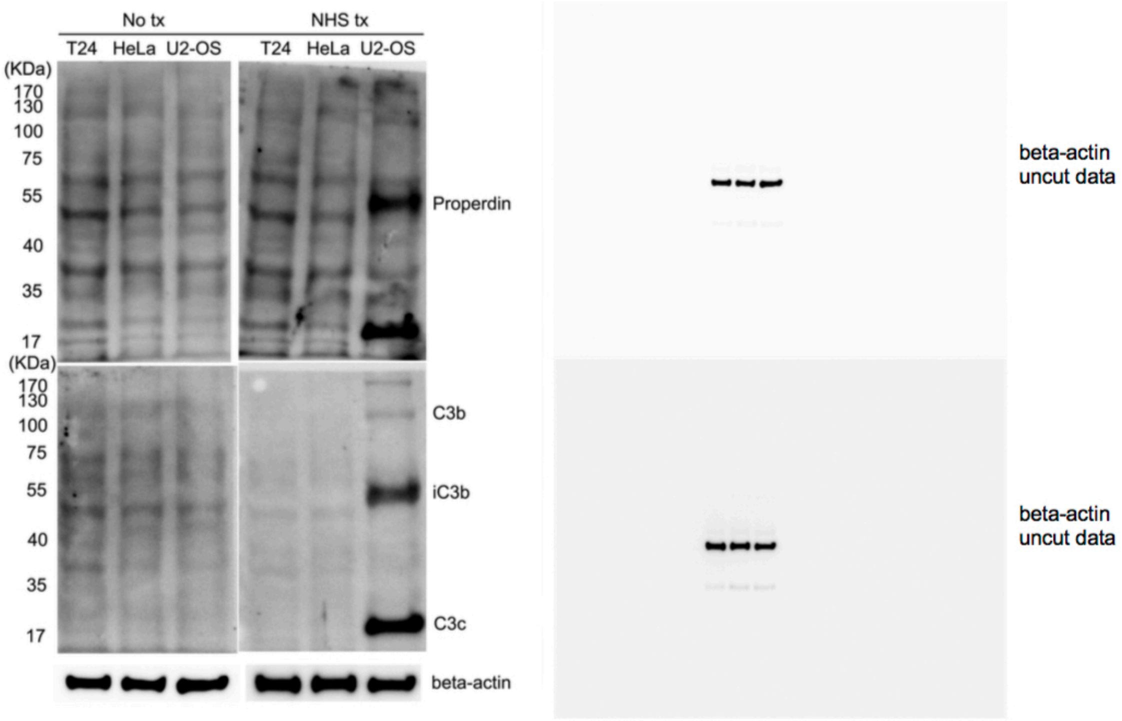

Figure S6. Western blot uncut membrane data of Figure 3B.

Figure S7.

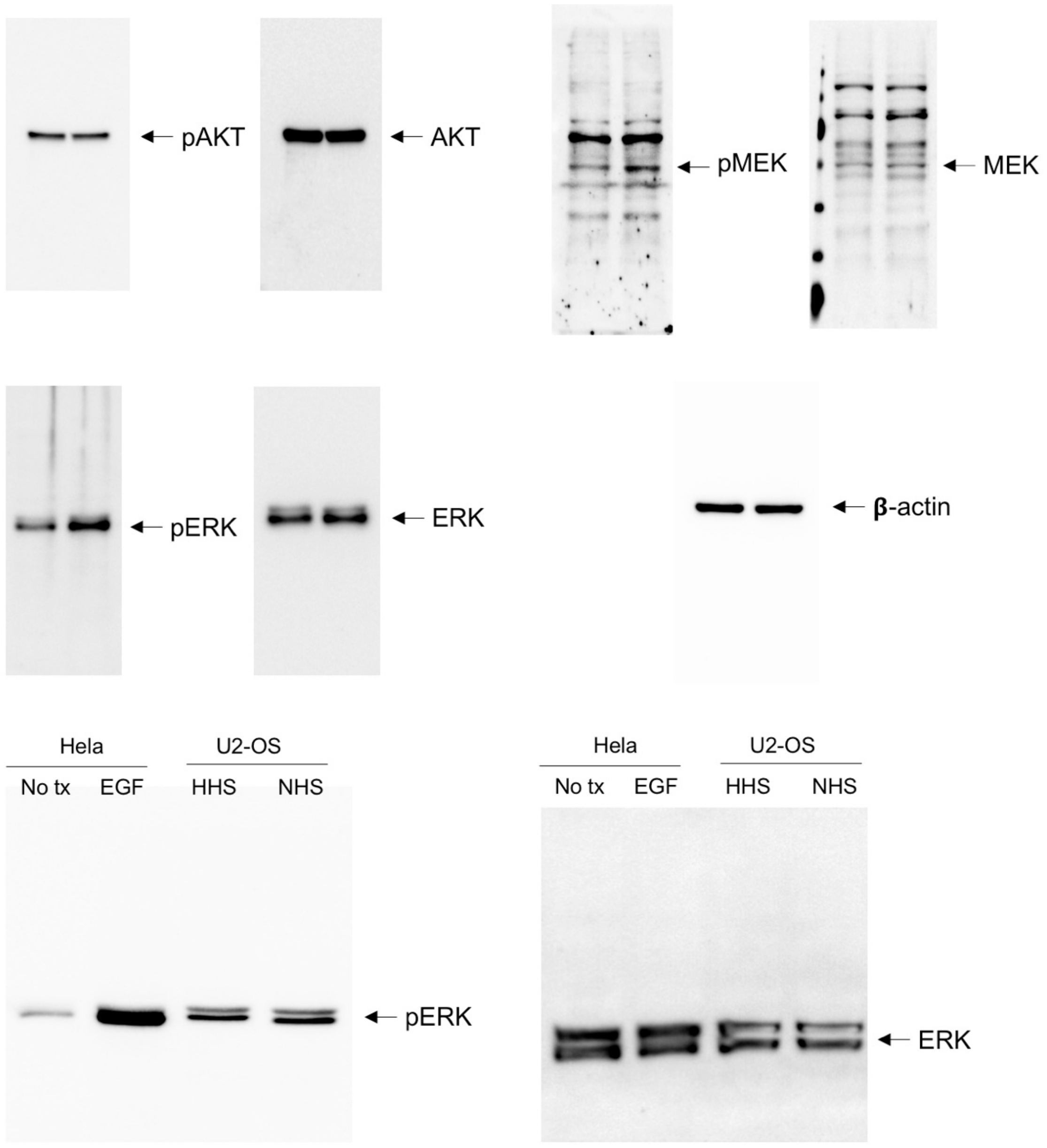

Figure S7. Western blot uncut membrane data of Figure 6A. Hela cells which treated with 20ng/ml of EGF was used as positive control for pERK. No tx: no treatment.
